# Single-phase transformerless nine-level inverter with voltage boosting ability for PV fed AC microgrid applications

**DOI:** 10.1038/s41598-022-16057-x

**Published:** 2022-08-04

**Authors:** Dhafer J. Almakhles, M. Jagabar Sathik

**Affiliations:** Renewable Energy Lab, College of Engineering, Prince Sultan University, Riyadh, Saudi Arabia

**Keywords:** Engineering, Electrical and electronic engineering

## Abstract

This letter presents a new single-stage common ground type nine-level (9L) switched-capacitor inverter topology with single-phase operation. The primary objective of this topology is to reduce the leakage current, voltage boosting, and maintain the voltage across the switched capacitors. The output voltage (v_o_) can be boosted up to two times the input voltage (v_in_). The various modes of operations are explained in detail, and the simulation results are provided to illustrate the effectiveness of the proposed topology. Next, the experimental results are obtained from a 500 W prototype setup and tested under different scenarios such as load variations, input voltage, and modulation index. Both simulation and experimental results have a good agreement regarding efficiency and performance. Finally, a detailed comparative study is performed with other recent 9L switched-capacitor inverters to prove the merits of the proposed topology.

## Introduction

In recent years, the switched capacitor inverters (SCIs) have been paid more attention due to their inherent voltage boosting capability and higher number of output voltage levels. Such inverters are more suitable for medium voltage applications, including distributed power generation systems^1^. The remarkable advantages of SCI topologies are: (1) lower number of active components, (2) floating capacitor (FC) voltages are either balanced by additional sensor/circuits or inherently balanced (so-called self-balanced), and (3) voltage boosting ability which further reduces the size or eliminates the front dc/dc converter for renewable energy applications. The two-level SCI has challenges such as high total harmonics distortion and large size of LC filter. To overcome these issues, the combination of switched-capacitor and multilevel inverter (SCMLI) is introduced in^1–3^ as multilevel inverters are very popular due to their reduced switch stress, better harmonic performance, lower losses, and reduced filter requirement^2–13^. A new generalized structure SCMLI topology with a higher number of output voltage levels is proposed in^2,3^. These topologies are suitable for applications with strict harmonic requirements. Still, the total switch count is high with a voltage gain of 1:*n* (*v*_*in*_:*v*_*out*_), and each capacitor voltage rating is equal to *v*_*in*._ There are several SCMLI topologies in the literature with a number of output voltage levels ranging from 4-level to generalized *n*-level. However, the number of output voltage levels in this paper is 9L, as found in^4–10^. These topologies have a common output voltage gain of 1:2 and need two floating capacitors (FCs) to generate a 9L output voltage waveform. However, each topology has its pros and cons. For example, more components are needed in^4^, whereas authors in^5^ have successfully reduced the switch count, and both the topologies produce the voltage gain of 1:2.

Another topology, which needs eight unidirectional switches and two RB-IGBTs, is proposed in^6^, and eight switches, two diodes, and three capacitors are used in^7^. A new topology with seven unidirectional switches, two diodes, and capacitors is used in^8^, but this topology also needs additional two RB-IGBT switches. However, in these topologies, the voltage gain is two, and most of the switch voltage rating is equal to the maximum output voltage. To reduce the voltage stress on the switches, two new topologies are presented in^9,10^ with voltage stress of *v*_*in*_, but the number of the semiconductor device is considerably high. Due to the leakage current, none of those mentioned above topologies are suitable for transformerless solar PV applications.

In recent years significant research efforts have been given in the development of transformerless inverter (TLI) topologies for photovoltaic application due to the elimination of leakage current^11–14^. A new five-level transformerless inverter with a reduced switch count is presented in^11^. However, this topology has a large filter requirement due to lower output voltage levels. To address this issue, a 9-level (9L) TLI is proposed in^12^ but does not have voltage gain, and the number of ON state switches is high at each level. Moreover, this topology's input voltage requirement is significantly higher due to reduced dc-bus utilization. Another novel 9L-TLI topology is proposed in^13^, which has full dc-bus utilization. The buck-boost converters are integrated with a series connection of four dc-link capacitors to achieve the nine-level output voltage. However, the dc-link capacitors are not balanced, leading to unsymmetrical stepped waveforms at the load. In this reference, it may be noted that the common connection between the load side ground terminal and the source side negative terminal eliminates the leakage current and is called the common ground connection^14^. Recent 9L SCMLI topologies with boosting are presented in^15–17^. However, they fail to reduce leakage current. Moreover, the topologies presented in^18–20^ share a common ground with eradicating leakage current. The^18^ presents a new common ground in the three-level inverter. The capacitor acts as a virtual dc source in the negative half cycle. If the capacitor fails, the topology will not generate the negative half cycle, and the entire system will fail. However, if any capacitor fails in the proposed topology, the magnitude of the output voltage and step size will be reduced, but the operation will continue.

Motivated by the above discussion, this paper presents a new common ground type (CGT) inverter with a reduced total power component. The proposed topology is an improved version of^10^. The proposed topology successfully reduces the number of power components, achieving a low voltage rating of FCs and low power loss with a maximum of ~ 97.2% of efficiency. It is important to note that the proposed topology reduces leakage current due to common grounding, which is missing in the existing 9L SCMLI inverter topologies^2–13^.

## Proposed TL-9L inverter topology

### Description of proposed topology

**Figure 1:**
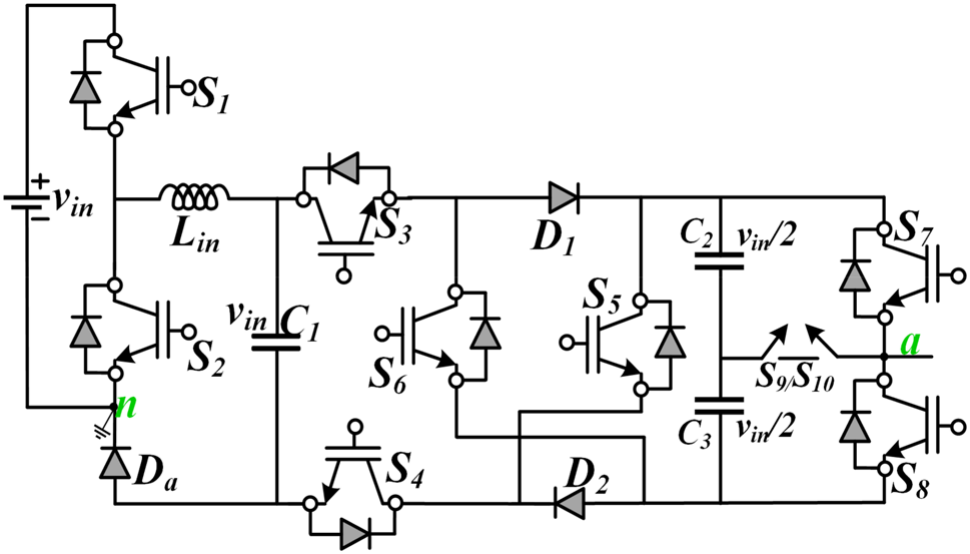
Proposed CGT-CSC 9L circuit diagram.

A detailed explanation of the circuit diagram and the modes of operations are discussed for the proposed TL-9L inverter. A detailed explanation of the circuit diagram and the modes of operations are discussed for the proposed CGT-T9L inverter. Figure 1 shows the circuit diagram of the proposed 9L inverter topology. It can be observed from Fig. 1 that it has a single dc source with three FCs (C_1_, C_2_, and C_3_) rated at voltage *v*_*in*_ /2 (C_2_, and C_3_) and *v*_*in*_, (C_1_). The proposed topology uses ten switches (S_1_, S_2_, S_3_, S_4_, S_5_, S_6_, S_7_, S_8_, S_9_, and S_10_) and three diodes (D_1_, D_2_, and D_a_). The capacitor C_1_ is connected in such a way that it gets charged when S_1_ is turned ON. The series-connected capacitors C_2_ and C_3_ are connected with switches S_7_, S_8,_ and S_9_/S_10_. The switch pairs S_7_, and S_8_ should not be turned ON simultaneously to avoid the short circuit of the series-connected capacitor branch. Likewise, the switch pairs (S_1_, S_2_), (S_3_, S_6_, S_4_), and (S_3_, S_5_, S_4_) should not be turned ON simultaneously. The midpoint of C_2_ and C_3_ is connected with S_9_ and S_10_. It may be observed that the switches S_7_, S_8,_ and S_9_/S_10_ form a T-type leg which also serves as one of the terminals of the load, i.e., node *a*. The load terminal is directly connected to the negative terminal of the dc source i.e., node *n*.

### Modes of operation with pulse generation scheme

**Figure 2:**
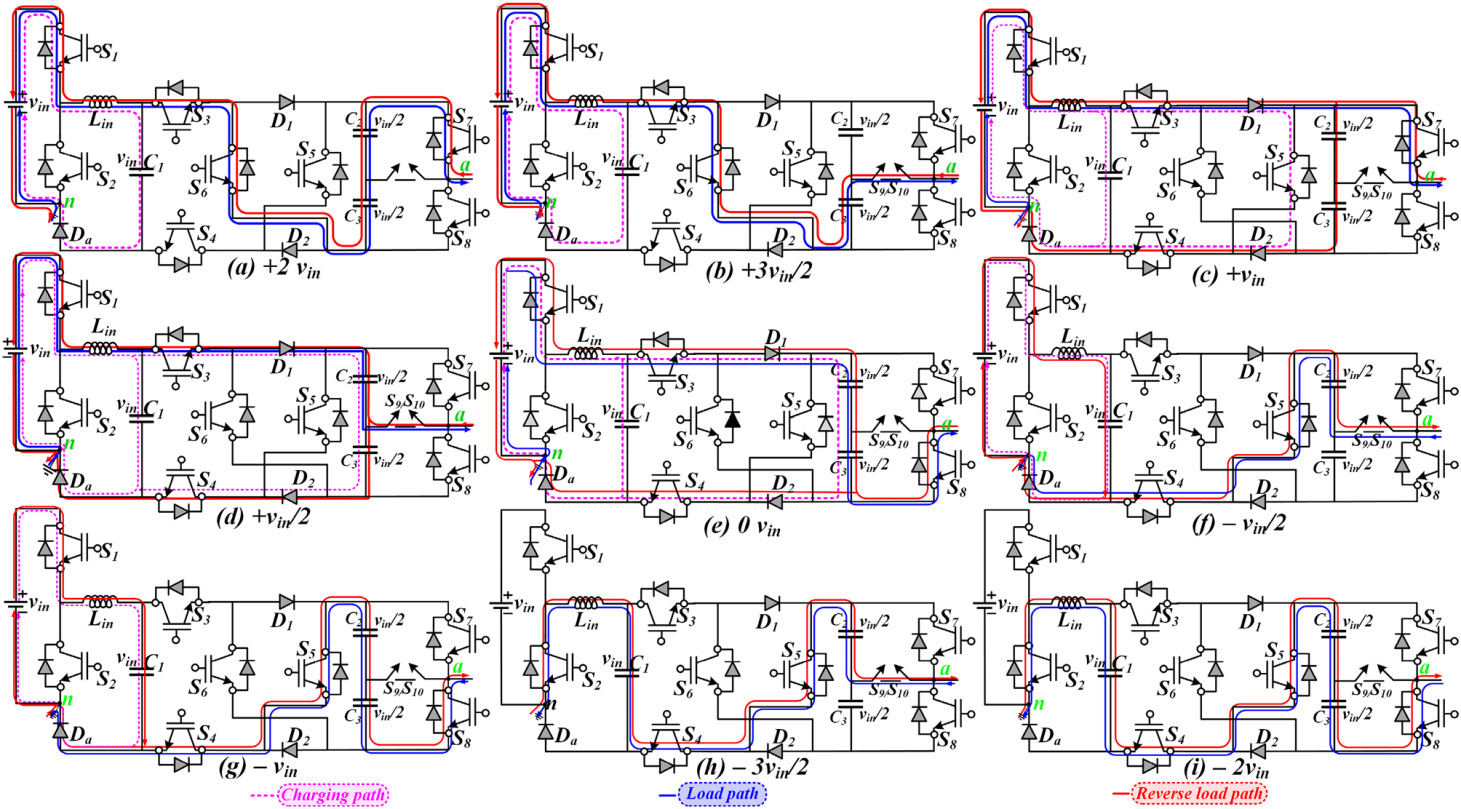
(**a**–**i**) Modes of operation of the proposed topology.

Figure 2a–i shows the sub-circuit diagram of the various modes of operation, showing the path of the charging current and load current. The capacitor C_1_ gets charged in all the positive levels and the first level of the negative half cycle. A few modes are explained below to understand the working principle of the whole topology.

In Fig. 2, three different color lines are shown, which illustrate the current path of the proposed circuit diagram during (1) the charging of the capacitors, (2) the current path for unity power factor, and (3) the current path in either lagging or leading power factor.

**Mode + *v***_***in***_**/2** In this mode, the FC C1 and the series-connected capacitor branch (C_2_ and C_3_) are charged simultaneously up to the *v*_*in*_. The switch S_9_/S_10_ is turned ON and the load voltage is equal to + *v*_*in*_/2, as shown in Fig. 2a.

- The closed path for charging C_1_-C_3_ is shown as: $v_{in} \to S_{1} \to \uparrow C_{1} \to S_{3} \to D_{1} \uparrow C_{2} \uparrow C_{3} \to D_{2} \to S_{4} \to D_{a} \to v_{in}$
- The closed current path for load is shown as: $v_{in} \to S_{1} \to S_{3} \to D_{1} \to D_{2} \to S_{9} S_{10} \to Load \to v_{in}$

**Mode + *v***_***in***_ In this mode, all the FCs charge simultaneously, and the load voltage equals + *v*_*in,*_ as shown in Fig. 2b.

- The closed path for charging of C_1_-C_3_ is shown as: $v_{in} \to S_{1} \to \uparrow C_{1} \to S_{3} \to D_{1} \uparrow C_{2} \uparrow C_{3} \to D_{2} \to S_{4} \to D_{a} \to v_{in}$
- The closed current path for load is shown as: $v_{in} \to S_{1} \to S_{3} \to D_{1} \to D_{2} \to S_{7} \to Load \to v_{in}$

**Mode + *3v***_***in***_***/2*** In this mode, FC C_3_ discharges, but FC C_1_ charges. The load voltage is equal to + 3 *v*_*in*_/2, as shown in Fig. 2c.

- The closed current path for charging of C_1_ is shown as: $v_{in} \to \uparrow C_{1} \to D_{a} \to v_{in}$
- The closed current path for load is shown as: $v_{in} \to S_{1} \to S_{3} \downarrow C_{3} \to S_{9} S_{10} \to v_{in}$

**Mode + *2v***_***in***_ In this mode, the FC C_2_ and C_3_ get discharged, and the load voltage is equal to + 2 *v*_*in,*_ as shown in Fig. 2d.

- The closed current path for charging of C_1_ is shown as: $v_{in} \to \uparrow C_{1} \to D_{a} \to v_{in}$
- The closed current path for load is shown below: $v_{in} \to S_{1} \to S_{3} \downarrow C_{3} \to S_{7} \to v_{in}$

**Mode + 0*v***_***in***_ During this mode, all the FCs are charging, and the load voltage is equal to + 0 *v*_*in,*_ as shown in Fig. 2e.

- The closed path for charging C_1_–C_3_ is shown below: $v_{in} \to S_{1} \to \uparrow C_{1} \to S_{3} \to D_{1} \uparrow C_{2} \uparrow C_{3} \to D_{2} \to S_{4} \to D_{a} \to v_{in}$

**Figure 3:**
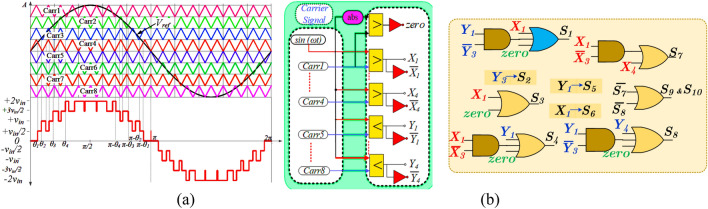
(**a**) Typical 9L output voltage waveform (**a**) sinusoidal PWM for 9L inverter and (**b**) PWM logic.

The explanation of the various modes for the negative half cycle is similar, and the corresponding turned-on switches for each voltage level and charging and discharging state of the capacitors are given in Table 1. Further, as shown in Fig. 2, the proposed topology can operate in both real and reactive power. The conventional level-shifted SPWM (LSPWM), as shown in Fig. 3a, and the PWM logic functions, as shown in Fig. 3b, are used in the proposed topology to generate the 9L output voltage. The reference voltage (*V*_*ref*_) is compared with the triangular carrier waveform (*Carr 1*–*Carr 8*) and produces the pulses. The simple logic functions are used to generate the desired pulses for the switches. Since the maximum output voltage of the proposed inverter is two times higher than the *v*_*in*,_ it generates the 9L stepped voltage waveform with each step of *v*_*in*_*/2.*

**Table 1:**
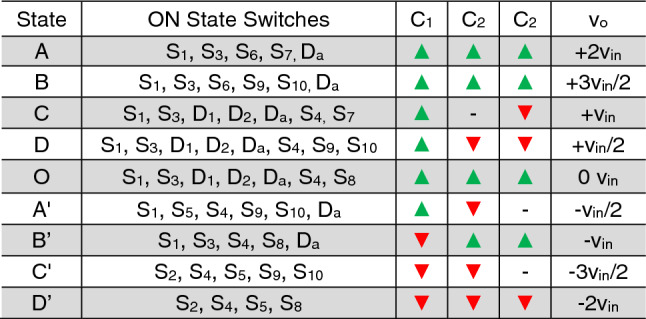
Switching sequence for proposed TL-9L inverter.

### Determination of capacitance and power analysis

#### Determination of capacitance

The proposed topology employs three capacitors, labeled C_1_, C_2_, and C_3_. These capacitors play a vital role in boosting the input voltage. Thus, the output voltage is equal to 2 *v*_*in*_, and each step has a voltage of *v*_*in*_/2. The switching capacitor C_1_ is charged to its maximum input voltage, while the flying capacitors C_2_ and C_3_ are charged to 50% of the input voltage. Consequently, the capacitance value selection of these capacitors is more critical to achieving the 9L output voltage. In addition, it affects the inverter's ripple loss, size, and total cost. As seen in Fig. 3a, the switched capacitor C_1_’s capacitance value was calculated using the longest discharging time of capacitors. The capacitances are estimated by using a maximum of 10% capacitor ripple voltage.

The capacitor C_1_ is discharged during all the negative levels (− 0.5 *v*_*in*_, − *v*_*in*_, − 1.5 *v*_*in*_, − 2 *v*_*in*_) as shown in Fig. 3a. The time duration t is estimated as follows,where 2π is the output voltage waveform's period. The predicted charge on the capacitor C1 under resistive load for the LDC period is:

$$\theta_{1} = \pi /10, \, \theta_{2} { = }\pi /5,\theta_{3} = 6\pi /20, \, \theta_{4} = 2\pi /5, \, \theta_{5} = \pi /2, \, \theta_{6} = 6\pi /10, \, \theta_{7} = 7\pi /10, \, \theta_{8} = 4\pi /5, \, \theta_{9} = 9\pi /10$$

1 $$Q_{{SC,_{C1 - R} }} = \int\limits_{{\theta_{10} }}^{2\pi } {I_{o} (t)} \, dt = 2\int\limits_{{\theta_{10} }}^{{\theta_{14} }} {I_{o} (t)} \, dt = 2\int\limits_{{\theta_{1} }}^{{\theta_{5} }} {I_{o} (t)} \, dt$$

2 $$Q_{{SC,_{C1 - R} }} = 2\int\limits_{\pi /10}^{\pi /2} {I_{o} (t)} \, dt = 2\left[ {\int\limits_{\pi /10}^{\pi /5} {I_{o} (t)} \, dt + \int\limits_{\pi /5}^{3\pi /10} {I_{o} (t)} \, dt + \int\limits_{3\pi /10}^{2\pi /5} {I_{o} (t)} \, dt + \int\limits_{2\pi /5}^{\pi /2} {I_{o} (t)} \, dt} \right]$$

The current load value of the proposed topology for the purely resistive load can be expressed as,

3 $$I_{O} (t) = \left\{ \begin{gathered} \frac{{{\text{v}}_{{{\text{in}}}} }}{{2}}{ ; }\frac{\pi }{10} \, \le \, t \, \le \, \frac{\pi }{5} \hfill \\ {\text{v}}_{{{\text{in}}}} { ; }\frac{\pi }{5} \, \le \, t \, \le \, \frac{3\pi }{{10}} \hfill \\ \frac{{{\text{3v}}_{{{\text{in}}}} }}{2}{ ; }\frac{3\pi }{{10}} \, \le \, t \, \le \, \frac{2\pi }{5} \hfill \\ {\text{2v}}_{{{\text{in}}}} { ; }\frac{2\pi }{5} \, \le \, t \, \le \, \frac{\pi }{2} \hfill \\ \end{gathered} \right.$$

From Eqs. (3) and (4), the charge on the capacitor C_1_ is estimated as,

4 $$Q_{{SC,_{C1} }} = \left[ {\frac{{{\text{v}}_{{{\text{in}}}} \pi }}{{{\text{R}}_{{{\text{LO}}}} }}} \right]$$

The optimal capacitance value of capacitors C_1_ when the load is entirely resistive may be calculated as follows:

5 $$C_{1optm - R} \ge \left[ {\frac{\pi }{{{\text{R}}_{{{\text{LO}}}} \times k \times \omega }}} \right]$$

When the load is resistive-inductive (RL), the load current is expressed as $I_{O} (t) = I_{mx} \sin (wt - \psi ) \,$.

At resistive-inductive (RL) loading conditions, the charge on capacitor C_1_ is approximated as follows:

6 $$Q_{{SC,_{C1 - RL} }} = \frac{{2I_{mx} }}{\omega }\left[ {\cos \left( {\frac{\pi }{10} - \psi } \right) - \sin (\psi )} \right]$$

The optimal capacitance value of capacitors C_1_ under resistive-inductive (RL) loading may be calculated as follows:

7 $$C_{1optm - RL} \ge \frac{{2I_{mx} }}{{k \times \omega \times {\text{v}}_{{{\text{in}}}} }}\left[ {\cos \left( {\frac{\pi }{10} - \psi } \right) - \sin (\psi )} \right]$$

The size of flying capacitors C_2_ and C_3_ are estimated as,

8 $$C_{2} = \frac{{I_{mx} }}{{f_{swg} \times \Delta {\text{V}}_{{{\text{c2}}}} }};C_{3} = \frac{{I_{mx} }}{{f_{swg} \times \Delta {\text{V}}_{{{\text{c3}}}} }}$$

where *I*_*mx*_ is the maximum load current, *f*_*s*_ is the switching frequency, and ΔV_C_ is the voltage ripple.

### Power loss analysis

Switching, conduction, driver circuit, and ripple loss are used to compute inverter power loss^19^. IGBT switching loss occurs when its anti-parallel diode is off (9)

9 $$E_{a} = E_{{IGBT,t_{ON} }} + E_{{Diode,t_{OFF} }} = \frac{1}{2}\left( {V_{CE} I_{o} t_{r} } \right) + \frac{1}{2}\left( {V_{F} I_{o} t_{r} } \right)$$

Switching loss during the IGBT is OFF, and Diode is ON as expressed in (10)

10 $$E_{b} = E_{{IGBT,_{{t_{OFF} }} }} + E_{{Diode,t_{ON} }} = \frac{1}{2}V_{CE} I_{o} \left( {t_{f} + t_{d(OFF)} } \right) + \frac{1}{2}\left( {V_{F} I_{o} t_{f} } \right)$$

The conduction losses are always high due to the long conduction period, and this can be calculated by using (11)

11 $$\begin{gathered} E_{{IGBT,_{ON} }} = I_{o}^{2} R_{CE(ON)} \left( {dTS - t_{r} - t_{d(ON)} } \right) \hfill \\ E_{{Diode,_{ON} }} = V_{F} I_{L} \left( {(1 - d)TS - t_{r} - t_{d(OFF)} } \right) \hfill \\ \end{gathered}$$

where ‘V_CE_’ is the collector and emitter voltage of IGBT, i.e. blocking voltage, 'V_F_' forward voltage of the diode, 'I_o_' is the load current, and 't_r_' rise time, t_d(ON)_ and t_d(OFF)_ is turn ON, and OFFF delay and '*t*_*f*_' is fall time of an IGBT. '*R*_*CE(on)*_' is the on-state resistance of an IGBT, '*d*' is the duty cycle of the IGBT, and '*T*_*s*_' is the switching period.

Further, the gate driver loss is very small and negligible. However, the calculation of the gate driver loss is given in (12)

12 $$E_{{IGBT,_{GD} }} = Q_{B} \times V_{BE} \times f_{s}$$

where the '*Q*_*B*_*'* is charge at the base terminal, 'V_BE_' biasing voltage to the IGBTs_,_ and *f*_*s*_ switching frequency. The energy loss across the capacitor during the charging is expressed as $E_{Cap} = \left( {{1 \mathord{\left/ {\vphantom {1 2}} \right. \kern-\nulldelimiterspace} 2}} \right)\left\{ {C \times \left( {\Delta V} \right)^{2} } \right\}$. C_2_ and C_3_ charge twice per half-cycle, whereas C_1_ charges four times. Average capacitor cycle loss is $E_{Rip} = 2f_{o} (E_{Cap} )$. Thus, the total across the capacitors in the complete cycle is $E_{Rip} = 2f_{o} (E_{C2} + E_{C3} ) + 4f_{o} (E_{C1} )$.

## Results and discussion

**Figure 4:**
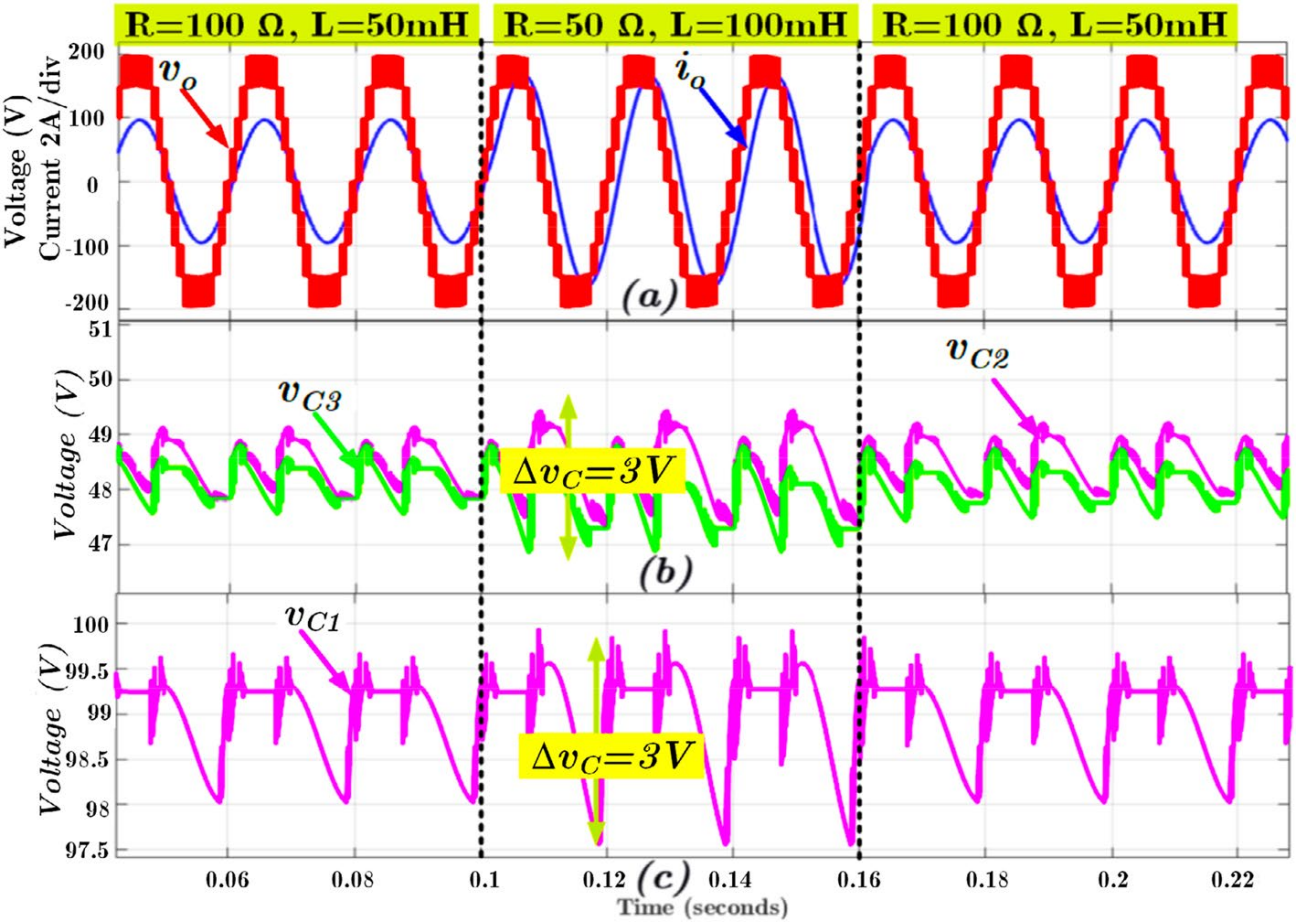
Simulation results of (**a**) output voltage and current; (**b**) voltages of the FCs C_2_ and C_3_, and (**c**) voltage of the FC C_1_.

**Figure 5:**
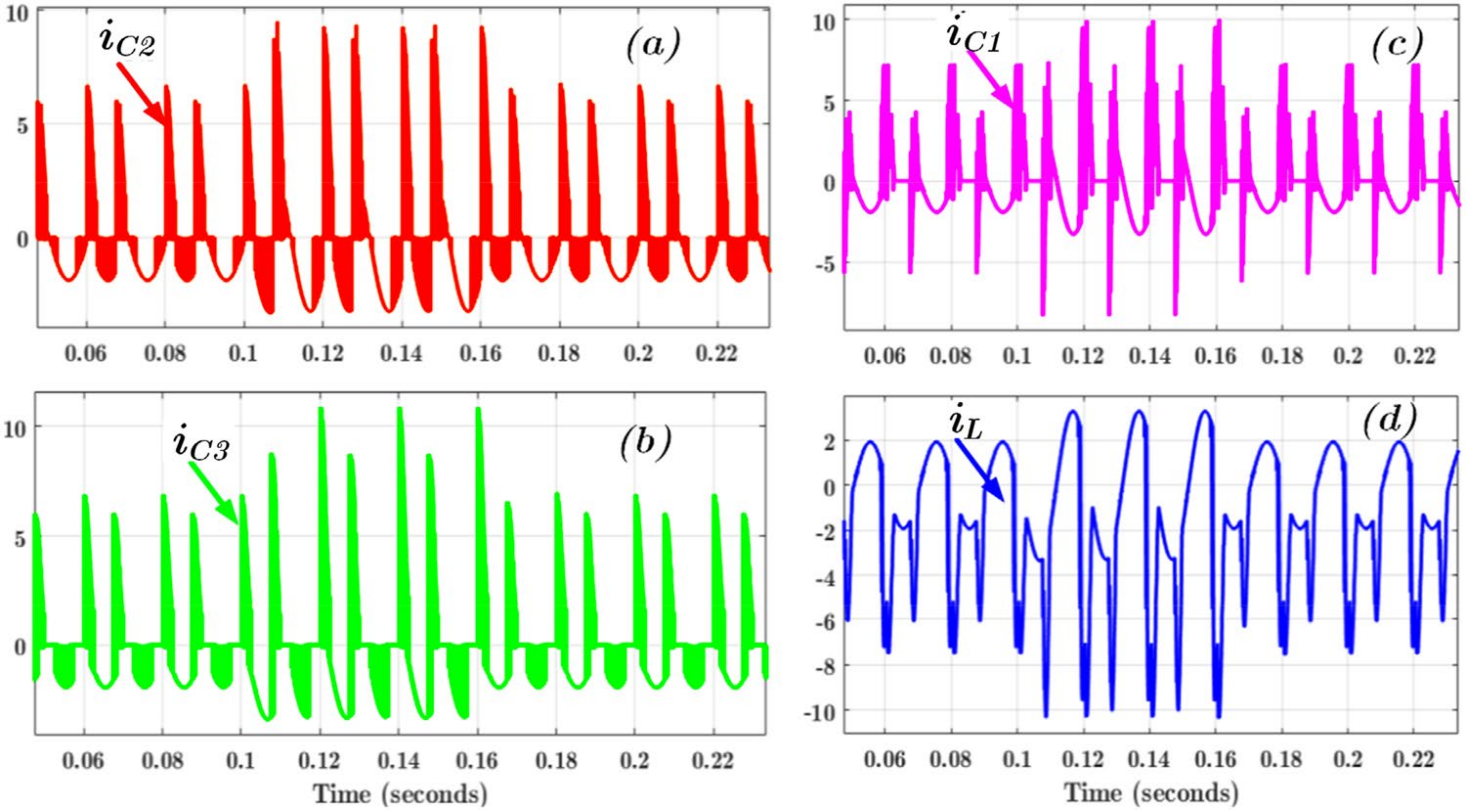
Simulation results during load changes (**a**)-(**c**) FCs current (*i*_*C1*_ − *i*_*C3*_) and (**d**) inductor current (*i*_*l,ind*_).

The performance of the proposed topology is validated in MATLAB / Simulink software tool and a laboratory-built hardware prototype. The input DC source voltage is kept at 100 V. The voltage and capacitance value of FC C_1_ is respectively 100 V and 2700 µF. Similarly, the voltage and capacitance values of FCs C_2_ and C_3_ are 50 V/2700 µF, respectively. The capacitance values are designed per the process given in^14^, where the maximum voltage ripple is chosen as less than 5% with a 2.5 kHz switching frequency. The two resistive-inductive loads are used with R = 100 Ω, L = 50 mH, and R = 50 Ω, L = 100 mH. The output voltage and current waveform with these two loads are given in Fig. 4a. The corresponding capacitor ripple voltage is given in Fig. 4b for C_2_ and C_3_ and Fig. 4c for C_1_. Further, the simulation result of each FCs current is shown in Fig. 5a–d, and the loop inductor current is shown in Fig. 5d.

**Figure 6:**
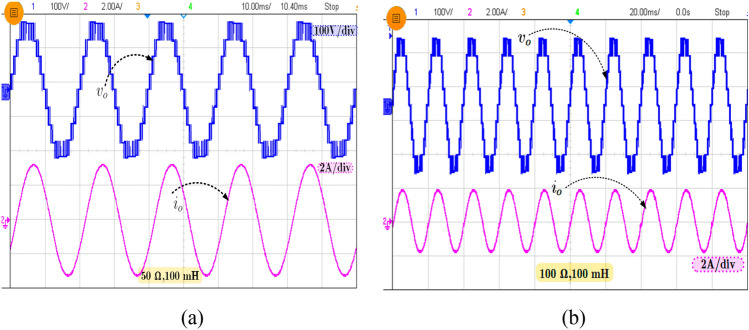
Experimental results showing the output voltage and current waveform for (**a**) R = 50 Ω, L = 50 mH and (**b**) R = 100 Ω, L = 50 mH.

For experimental validation for 500 W prototype model was rigged up with the DC Source taken from a three-phase rectifier (600 V, 100A), the capacitors C_1_-2200 µF/100 V (ALS30A222DA100) and C_2_, C_3_-1700 µF/50 V (SLPX332M050C1P3). A little higher value of capacitance is chosen due to practical considerations like suppressing the effect of unnecessary parasitic wiring inductance. Here, the power electronics devices are chosen as IGBTs (600 V, 73 A) SKM75GB63D from Semikron. The diode (200 V, 30 A) HER3003 from DC Components is used where only a single diode is used in the proposed topology as given in Table 2. A notable drawback of the switched capacitor circuits is the high inrush current. In this paper, a current limiting inductor is used to reduce the inrush current. The inductor size is small and can limit the inrush current to an allowable value^14^. The mathematical expression for the current limiting inductor is given in (13).

13 $$i_{l,in} = \frac{1}{2}\sqrt {\frac{{C_{f} }}{{L_{in} }}\Delta } V_{Cf}$$

where the *i*_*l,in*_ is the maximum inrush current or loop current during the charging of the FCs. *L*_*in*_ is inductance value and *C*_*f*_ is FC capacitance value. *i*_*c*_ is charging current i.e. FC current which is usually four to five times higher than the load current. For the suppression of inrush current the loop inductor value is chosen as ~ 20 A for *L*_*l,in*_ = 40 μH based on (1). The experimentally obtained output voltage and current waveforms for R = 50 Ω, L = 100 mH (peak current (*I*_*peak*_) = ∼ 3.3 A) with power factor of 0.85 and R = 100 Ω, L = 50 mH (*I*_*peak*_ = ∼ 1.9 A) with power factor of 0.99 is presented in Fig. 6a,b, respectively, and they confirm that the peak of the output voltage is 200 V which is two times higher than the input voltage. Most of the loads are dynamic behavior, so it is necessary to evaluate the proposed topology for different loading condition.

**Table 2:** Experimental parameters.

S. No.	Description	Specification
1	IGBTs	SKM 75GB063D 600 V/75 A
2	Capacitors	SLPX332M050C1P3/1700 µF/50 V ALS30A222DA100/2200 µF/100 V
3	Diode	HER3003 200 V/30A
4	Input/output voltage	100 V/200 V
5	Load R and RL values	R = 50 Ω, L = 100 mH/R = 100 Ω to 55 Ω R = 100 Ω, L = 50 mH

**Figure 7:**
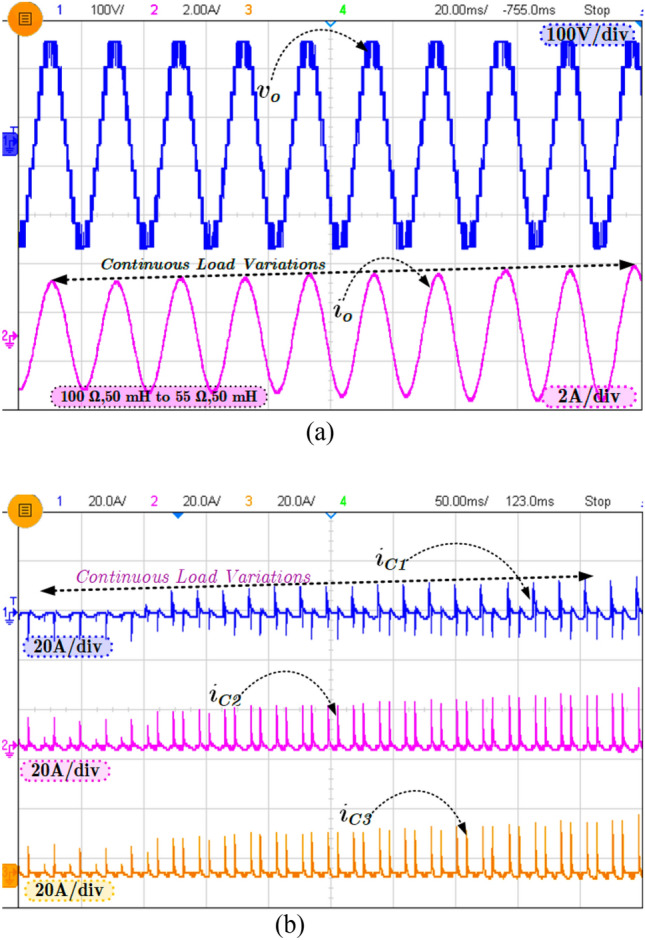
Experimental results (**a**) Continuous load change and (**b**) corresponding capacitors (C_1_–C_3_) current.

Figure 7a shows the continuous load change from R = 100 Ω to 55 Ω with constant L = 50 mH, and in Fig. 7b, the capacitor current for load change is presented. As explained earlier, in SCMLI topologies, the inrush current is a big challenge addressed in the proposed topology using the loop inductor. The current flowing through the FCs is presented in Fig. 7b.

**Figure 8:**
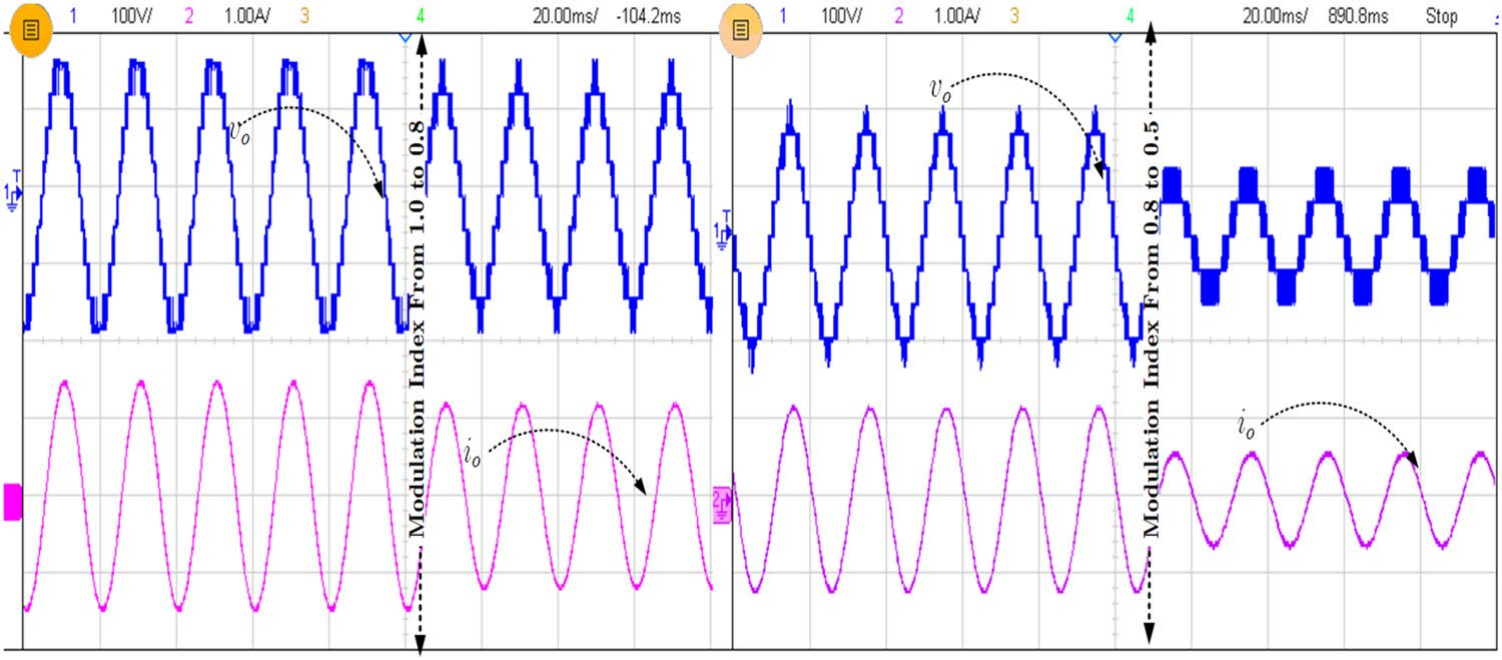
Experimental results of modulation index variation from 1.0 to 0.5.

**Figure 9:**
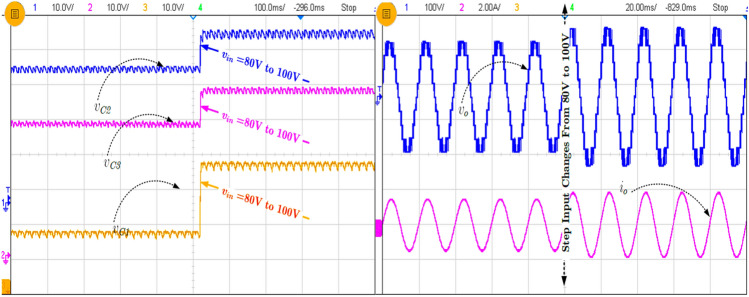
Experimental result of step input change from 80 to 100 V.

**Figure 10:**
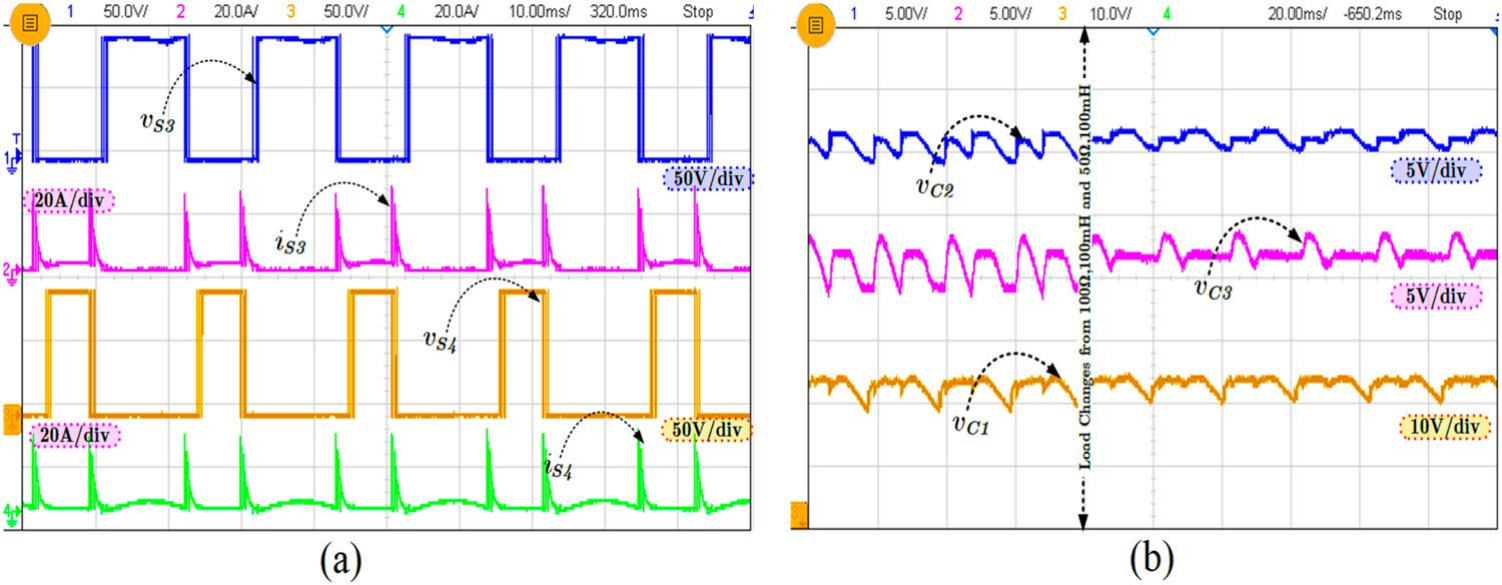
Experimental result of (**a**) the switch voltage and current and (**b**) capacitor voltages (C_1_–C_3_).

It can be observed from Fig. 7b that the inrush current or the FC current is significantly reduced in the proposed topology but the inrush current is a little higher than the calculated value. However, the inductor (L_in_) reduced the maximum inrush current four times less than the without inductor current. In order to show the performance of the proposed topology during modulation, index variations are tested, and the corresponding waveform is shown in Fig. 8. Further, the various experimental results for step input change (80–100 V) with FCs voltages are shown in Fig. 9. The blocking voltage and current of switches (S_3_ and S_4_) as shown in Fig. 10a, and capacitor voltage for load changing is presented in Fig. 10b. The voltage stress on the switches is equal to the *v*_*in*_, whereas other topologies presented in Table 3 show the voltage stress is two and four times higher than the *v*_*in*_. Most of the SCMLI topology with boosting ability circuits have higher current stress than the conventional inverter. It can be limited by inserting the small inductor in the capacitor charging path, but it is worth mentioning that the voltage boosting ability without any additional circuit is achieved. The comparison of the proposed TL-9L inverter topology with other recent 9L SCMLIs is presented in Table 3. It can be observed that the number of power components count is considerably low in the TL-9L inverter. However, it may also be observed from Table 2 that a few existing topologies have fewer switches compared to the proposed topology.

**Table 3:** Comparison of proposed topology with other recent nine-level inverter topologies.

Refs.	N_SW_	N_GD_	N_D_	N_FC_	G	FC_Max_	NFC_Max_	I_rc_	Leakage current	Boosting feature	MSVp.u	Efficiency (η) %
^2^	13	13	-	3	1:4	v_in_	3	Yes	Yes	Yes	4v_in_	85.9@ < 100 W/1 kHz
^3^	10	10	1	3	1:4	v_in_	3	Yes	Yes	Yes	4v_in_	91.6@260 W/50 Hz
^4^	10	8	1	2	1:2	0.5 v_in_	2	Yes	Yes	Yes	2v_in_	96.0@1 kW/50 Hz
^5^	8	8	1	2	1:2	v_in_	1	Yes	Yes	Yes	2v_in_	96.47@333.33 W/50 Hz
^6^	10	9	–	2	1:2	0.5 v_in_	2	Yes	Yes	Yes	2v_in_	96@1 kW/50 Hz
^7^	8	8	2	3	1:2	v_in_	2	Yes	Yes	Yes	2v_in_	95.6@0.5 kW/50 Hz
^8^	9	9	2	2	1:2	v_in_	2	Yes	Yes	Yes	2v_in_	96.2@1 kW/50 Hz
^9^	11	10	–	2	1:2	0.5 v_in_	2	Yes	Yes	Yes	v_in_	NA
^10^	10	9	2	2	1:2	0.5 v_in_	2	Yes	Yes	Yes	v_in_	97.12@400 W/50 Hz
^11^	8	8	–	1	1:1	0.25 v_in_	1	Yes	Yes	No	v_in_	98@1 kW/50 Hz
^12^	10	10	-	1	1:0.5	0.25 v_in_	1	No	Yes	No	v_in_/2	94.15%@400 W/50 Hz
^13^	12	11	2	4	1:2	v_in_	3	No	Yes	Yes	3v_in_	94.90%@500 W/50 Hz
^14^	9	7	8	2	1:1	0.25 v_in_	2	No	No	Yes	v_in_	97.7%@187.52 W/50 Hz
^15^	11	10	–	2	1:2	0.5 v_in_	2	Yes	Yes	Yes	v_in_	NA
^16^	10	10	1	2	1:4	2v_in_	2	Yes	Yes	Yes	4v_in_	94.3%@500 W/50 Hz
^17^	12	11	–	3	1:2	v_in_	2	Yes	Yes	Yes	v_in_	95%@500 W/50 Hz
^19^	9	8	1	3	1:2	v_in_	3	No	No	Yes	2v_in_	97.5%@1.2 kW/400 Hz
^20^	14	14	–	4	1:4	v_in_	4	No	No	Yes	4v_in_	96.54%@900 W/50 Hz
Prop	10	9	3	3	1:2	v_in_	1	No	No	Yes	v_in_	96.0@ ~ 500 W/50 Hz

N_SW_/N_GD_/N_D_/N_FC_—number of switches/gate driver/diode/floating capacitor, T_Comp_—total component count, F_CMax_—maximum voltage rating of FC, NFC_Max_—Number of FCs with maximum voltage rating, MSV_p.u_—Maximum Standing Voltage, I_rc_—Inrush Current.

**Figure 11:**
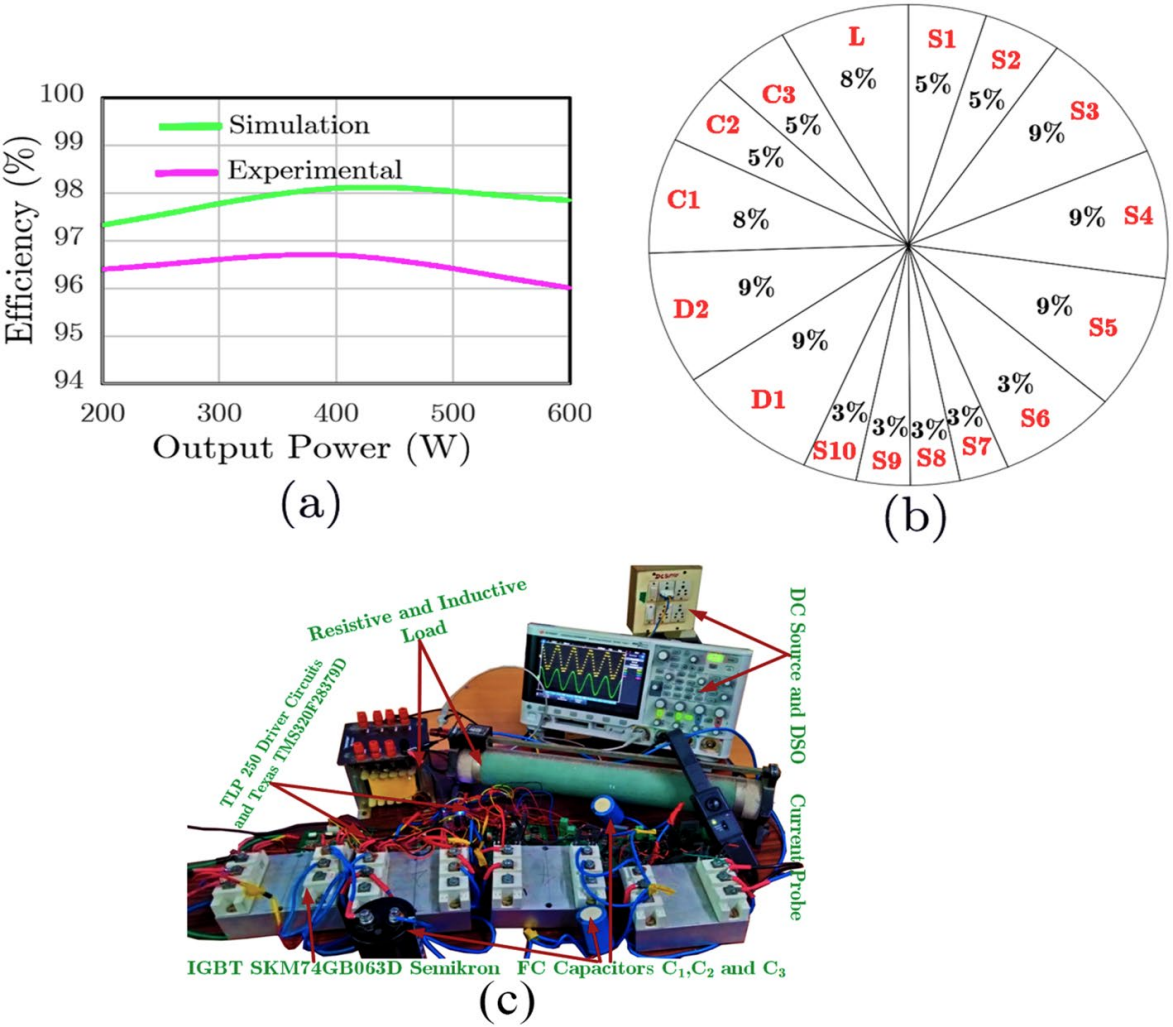
Power Loss (**a**) component loss breakdown using PLECS for 500 W, (**b**) Efficiency curve for unity power factor and (**c**) Scaled experiment photo.

However, the proposed topology is addressed the leakage current problem entirely as it is of common ground type and these features are not available in any other 9L inverters presented in the given literature. Apart from this, the proposed topology can address the inrush current issue despite being a boost-capable switched capacitor type. It may be noted that none of the existing switched capacitor type 9L inverters^3–17,19,20^ have this advantage. The power loss breakdown of the individual components of the proposed topology is given in Fig. 11a. Due to the charging current, the losses are high in FCs and the IGBTs carrying the charging current. The simulation efficiency of the proposed topology is 97.4%, with a total loss of 13 W for ~ 500 W output power. However, in the experimental setup, the measured efficiency is ~ 95.8% for the unity power factor, as shown in Fig. 11b, with an approximate total power loss of ~ 21 W. The experimental efficiency is measured using the Fluke 434-II power quality meter. The photo of the scaled-down experiment setup is shown in Fig. 11c.

## Conclusion

This paper has presented a new TL-9L inverter topology width that reduced the total number of power components. The discussion confirms that the proposed topology requires a lower number of power components. The performance of the proposed topology is analyzed in both simulation and scaled experimental setup.

The simulation and experimental results are well in good agreement. The various results have proved the proposed topology has self-voltage balancing and boosting ability. Also, the proposed topology can withstand any sudden changes at the load side or input voltage changes. The power loss breakdown is given for 500 W with maximum efficiency of ~ 95.8%. The comparison between the proposed topology and similar recently developed topologies also shows the merits of the proposed topology in terms of component count and efficiency, which can consider a great advantage with respect to the available solutions for PV fed AC Microgrid Applications.

## Data Availability

The datasets analyzed during the current study are available from the corresponding author on reasonable request.
